# Monocular Visual Position and Attitude Estimation Method of a Drogue Based on Coaxial Constraints

**DOI:** 10.3390/s21165673

**Published:** 2021-08-23

**Authors:** Kedong Zhao, Yongrong Sun, Yi Zhang, Hua Li

**Affiliations:** Navigation Research Center, College of Automation Engineering, Nanjing University of Aeronautics and Astronautics, Nanjing 211106, China; sunyr@nuaa.edu.cn (Y.S.); zynuaa@nuaa.edu.cn (Y.Z.); huali@nuaa.edu.cn (H.L.)

**Keywords:** monocular vision, aerial drogue, position and attitude estimation, circular feature, duality

## Abstract

In aerial refueling, there exists deformation of the circular feature on the drogue’s stabilizing umbrella to a certain extent, which causes the problem of duality of position estimation by a single circular feature. In this paper, a monocular visual position and attitude estimation method of a drogue is proposed based on the coaxial constraints. Firstly, a procedure for scene recovery from one single circle is introduced. The coaxial constraints of the drogue are proposed and proved to be useful for the duality’s elimination by analyzing the matrix of the spatial structure. Furthermore, we came up with our method, which is composed of fitting the parameters of the spatial circles by restoring the 3D points on it, using the two-level coaxial constraints to eliminate the duality, and optimizing the normal vector of the plane where the inner circle is located. Finally, the effectiveness and robustness of the method proposed in this paper are verified, and the influence of the coaxial circle’s spatial structure on the method is explored through simulations of and experiments on a drogue model. Under the interference of a large amount of noise, the duality elimination success rate of our method can also be maintained at a level that is more than 10% higher than others. In addition, the accuracy of the normal vector obtained by the fusion algorithm is improved, and the mean angle error is reduced by more than 26.7%.

## 1. Introduction

Aerial refueling technology can significantly extend the endurance of aircraft, which is of great importance in strategic or tactical aviation operations [1]. It is well known at present that there are two kinds of aerial refueling system: the probe-and-drogue refueling system pioneered by Flight Refueling Ltd. [2] and the Flying Boom refueling system developed by Boeing [3]. Due to the economy and flexibility of the probe-and-drogue refueling method and its adoption by many countries, its use is advisable for autonomous aerial refueling [4]. In a docking situation, the principal issue is how to accurately and quickly measure the relative position and attitude between the probe (from the receiving aircraft) and the drogue (from the tanker) during the end game [4,5,6]. Monocular vision is the most popular and fastest-growing method and has been extensively studied by researchers owing to its feasibility, ease of calibration, low cost, passive nature, and large effective field of view [7,8]. Therefore, it is a common navigation method for the AAR docking stage [1,4,5].

Typical geometric features used for pose estimation include points, lines, and circles in vision-based relative pose measurements. Whether in theoretical research [9,10,11,12] or application [13,14,15,16], scholars at home and abroad have done a lot of research. Compared with other features, circular features are common and widespread in the aviation field. Circular features have a high anti-interference ability with respect to occlusion owing to their geometric characteristics. It is an accepted and advantageous method in aeronautics and astronautics to obtain the navigation information by using the circular features of the target to accomplish the task of the aircraft and spacecraft [17,18].

The shape of a drogue is a typical body of revolution (BOR) that contains many coaxial circular features. However, with a change in aircraft speed, the stabilizing umbrella of the drogue is in different opening and closing states, which leads to the deformation of the circular features to some extent during the refueling process. It is difficult to obtain in real time the effective radius of circular features for monocular vision pose estimation. Only the shape of the circle near the oil joint is fixed, which is called the ‘inner circle’ of the drogue in this paper. The 5 degrees of freedom (DOF) pose of the drogue can be estimated by the inner circle.

Nonetheless, there exists the problem of a dual solution for the pose estimation by a single circular feature, that is, there are two sets of the circle center’s positions and the normal vectors of the circle plane [19]. If and only if the optical center and the target circle form a cone is there a unique solution.

To eliminate the duality, in References [18,20] the authors proposed to construct a reference point in a plane or space and used the invariance in the Euclidean distance to the circle center as a constraint to select the correct pose. However, this method relies on a known point feature; in Reference [21], the constraint angle from a motion reconstruction was utilized to eliminate the duality, that is, the space angle does not change with the motion of the rigid body, but it requires dense point features in the scene for reconstruction. For the case of multiple circular features, the authors suggested that the pose estimation of the target be constrained by multiple parallel circular or cylindrical features [19,22]. The implicit condition is that the radius of all coaxial circles is known, which is not satisfied in the drogue case. The features of a double planar circle were applied to estimate the pose of the target in Reference [23] and calibrate the camera parameters in References [24,25]. In addition, stereo vision was introduced to the pose estimation by providing additional information to the scene reconstruction in References [26,27].

All of the methods mentioned above are limited by specific requirements and special conditions, such as the prerequisite of all information about the coaxial circles, the introduction of auxiliary equipment other than a monocular camera, the existence or installation of special structures that can be recognized, or the need for many features for reconstruction. Notwithstanding, it is not possible or at least difficult to install an artificial mark on the drogue and introduce other auxiliary equipment into the airborne monocular vision system for visual navigation in AAR or an Actively Stabilized Refueling Drogue System (ASRDS).

Aiming at solving the problem that only a single circular feature known in advance can be directly used for pose estimation, while the others are deformed to a certain extent during air refueling, a monocular visual position and attitude estimation method of a drogue based on coaxial constraints is proposed in this paper. It makes full use of multiple circular features of the drogue itself, and the proposed coaxial constraints can effectively eliminate the duality of solutions. Moreover, the accuracy of the target’s normal vector, optimized by fusing multiple circular features, is greatly improved.

The paper is organized as follows. Section 2 presents the method for scene recovery from a circular feature. In Section 3, we propose the coaxial constraints and prove them. The position and attitude estimation algorithm based on the coaxial constraints is presented in Section 4. Section 5 presents the simulations and experiments conducted to evaluate the proposed methodology. Section 6 presents the conclusions.

## 2. Scene Recovery from a Circle

### 2.1. Projection of a Circle

Define the world coordinate system, camera coordinate system, and image coordinate system of the object as $O_{w}-X_{w}Y_{w}Z_{w}$, $O_{c}-X_{c}Y_{c}Z_{c}$, and $O_{I}-UV$, respectively. Let ${\tilde{x}}^{\left(w\right)}={\left(x_{w},y_{w},z_{w},1\right)}^{T}$ denote the 3D homogeneous coordinates of a 3D point in the world coordinate system, and let ${\tilde{x}}^{\left(I\right)}={\left(u,v,1\right)}^{T}$ be the homogeneous coordinates of its projection in the image coordinate system. The projection from the world coordinate system to the image can be described as:

(1) $$\begin{array}{ccc} z_{c}{\tilde{x}}^{\left(I\right)}=Kx^{\left(c\right)}=K[R|t]{\tilde{x}}^{\left(w\right)} & with & K=\left[\begin{array}{ccc} f_{u} & 0 & u_{0}\\ 0 & f_{v} & v_{0}\\ 0 & 0 & 1 \end{array}\right] \end{array}$$

where $z_{c}$ is a scale factor (with a projection depth of x); K is the camera intrinsic matrix, with the focal length $\left(f_{u},f_{v}\right)$ and the principle point $\left(u_{0},v_{0}\right)$; R and t are the rotation matrix and the translation vector from the world coordinate system to the camera coordinate system, respectively; and $x^{\left(c\right)}={\left(x_{c},y_{c},z_{c}\right)}^{T}\in {\mathbb{R}}^{3}$ denote the non-homogeneous coordinates of the corresponding 3D point in the camera coordinate system.

Without loss of generality, all the discussions in this paper assume that the camera intrinsic matrix K has been calibrated. We establish a world coordinate system where the *x*–*y* plane lies on the plane of the circle and the *z*-axis of the coordinate system is perpendicular to the plane and faces away from the camera. A point $\begin{array}{ccc} {\tilde{x}}_{p}{}^{\left(w\right)}={\left(x_{p},y_{p},z_{p},1\right)}^{T} & with & z_{p}=0 \end{array}$ on the circle P of which the center homogeneous coordinates are ${\left(x_{o},y_{o},0,1\right)}^{T}$ with a radius of $r_{0}$ can be expressed by the following formula.

(2) $$\left\{\begin{array}{l} \begin{array}{ccccc} {\left({\tilde{x}}_{p}{}^{\left(xy\right)}\right)}^{T}P{\tilde{x}}_{p}{}^{\left(xy\right)}=0 & with & {\tilde{x}}_{p}{}^{\left(xy\right)}={\left(x_{p},y_{p},1\right)}^{T} & and & z_{p}=0 \end{array}\\ P=\left[\begin{array}{ccc} 1 & 0 & -x_{o}\\ 0 & 1 & -y_{o}\\ -x_{o} & -y_{o} & x_{o}{}^{2}+y_{o}{}^{2}-r_{0}{}^{2} \end{array}\right] \end{array}\right.$$

Combining Equation (1) with Equation (2), the projection of the circle in the image coordinate system is derived as:

(3) $$\left\{\begin{array}{l} {\left(\tilde{x}{}^{\left(I\right)}\right)}^{T}Q{\tilde{x}}^{\left(I\right)}=0\\ Q=\mu {\left(K\left[\begin{array}{ccc} r_{1} & r_{2} & t \end{array}\right]\right)}^{-T}P{\left(K\left[\begin{array}{ccc} r_{1} & r_{2} & t \end{array}\right]\right)}^{-1} \end{array}\right.$$

Denote $H=K\left[\begin{array}{ccc} r_{1} & r_{2} & t \end{array}\right]$. Q can be rewritten as $Q=\mu H^{-T}PH^{-1}$, where μ is a scale factor and equal to $z_{c}{}^{2}$. $r_{1}$ and $r_{2}$ are the first and second columns, respectively, of the rotation matrix R, and t is the translation vector.

Since Q is a symmetric matrix, it forms an elliptical cone by the optical center of the camera and the spatial circle under the conditions of the principles and the technique of imaging. Without considering the degradation that occurs when the projection of the circle becomes a line, the projection of the circle is an ellipse corresponding to the intersection of the image plane and the elliptical cone. The projection of the spatial circle on the image plane satisfies the ellipse constraint, which is consistent with the conclusion [25].

### 2.2. Position and Attitude Estimation from a Circle

The solution of a pose estimation problem from one circle is the position of the circle center $O^{\left(c\right)}={\left(x,y,z\right)}^{T}$ and the normal vector $n^{\left(c\right)}={\left(n_{1},n_{2},n_{3}\right)}^{T}$ of the plane where the circle is located in the camera coordinate system due to the rotational symmetry of the circle feature. The normal vector is equivalent to the direction of the *z*-axis of the world coordinate system in the camera coordinate system.

When the camera intrinsic matrix K has been calibrated, it is easy to transfer the image coordinates ${\tilde{x}}^{\left(I\right)}$ with $K^{-1}$. For the convenience of derivation, let $K=I_{3}=diag\left(1,1,1\right)$. Then, the matrix H can be rewritten and the inverse of the matrix can be derived as:

(4) $$\left\{\begin{array}{l} H=K\left[\begin{array}{ccc} r_{1} & r_{2} & t \end{array}\right]=R\left[e_{1},e2,R^{T}t\right]\\ H^{-1}=\left[e_{1},e_{2},s\right]R^{T} \end{array}\right.$$

where $e_{1}={\left(1,0,0\right)}^{T}$, $e_{2}={\left(0,1,0\right)}^{T}$, and $s={\left(s_{1},s_{2},s_{3}\right)}^{T}$. R, t, and s follow the rule $R^{T}t=s_{3}{}^{-1}\left[\begin{array}{c} -s_{1}\\ -s_{2}\\ 1 \end{array}\right]$ with $s_{3}\neq 0$. Combined with Equation (4), it can be derived from Equation (3) that:

(5) $$\begin{array}{ccc} Q=\mu RMR^{T} & \mathrm{with} & M=\left[\begin{array}{ccc} 1 & 0 & s_{1}\\ 0 & 1 & s_{2}\\ s_{1} & s_{2} & s_{1}{}^{2}+s_{2}{}^{2}-{\left(s_{3}r_{0}\right)}^{2} \end{array}\right] \end{array}$$

Under perspective geometry, the parameter matrix of a projected ellipse and the parameter matrix of the spatial circle are similar. According to Reference [28], the analytical solutions of position and attitude can be settled based on the theorem that if two matrices are similar, they have the same eigenvalues. Four sets of circle centers and normal vectors can be obtained by the Eigen-decomposition of the matrix Q, as:

(6) $$\begin{array}{l} o^{\left(c\right)}=-\omega_{1}r\sqrt{-\frac{\lambda_{3}\left(\lambda_{1}-\lambda_{2}\right)}{\lambda_{1}\left(\lambda_{1}-\lambda_{3}\right)}}v_{1}-\omega_{2}r\sqrt{-\frac{\lambda_{1}\left(\lambda_{3}-\lambda_{2}\right)}{\lambda_{3}\left(\lambda_{3}-\lambda_{1}\right)}}v_{3}\\ n^{\left(c\right)}=-\omega_{1}\sqrt{-\frac{\lambda_{1}-\lambda_{2}}{\lambda_{1}-\lambda_{3}}}v_{1}-\omega_{2}\sqrt{-\frac{\lambda_{3}-\lambda_{2}}{\lambda_{3}-\lambda_{1}}}v_{3} \end{array}$$

where $\omega_{1}=\pm 1,\omega_{2}=\pm 1$. $\lambda_{i}$ are the eigenvalues of the matrix Q and $v_{i}$ are the corresponding eigenvectors. According to the constraint that the *z*-axis of the camera coordinate system points to the target, two sets of solutions can be eliminated. The remaining two sets of solutions are the duality problems for the position and attitude estimation by a single circular feature under normal circumstances.

## 3. The Coaxial Constraints on a Drogue

### 3.1. Analysis of a Drogue

During the docking stage of autonomous aerial refueling, the control system that controls the motion state of the tanker and receiver, such as position, attitude, and speed, is extremely dependent on the relative pose between the drogue and the receiver obtained by the visual navigation system. In addition, the actively stabilized refueling drogue system (ASRDS) for stabilizing the drogue also relies on visual navigation to monitor the position and attitude between the drogue and the probe in real time.

**Figure 1:**
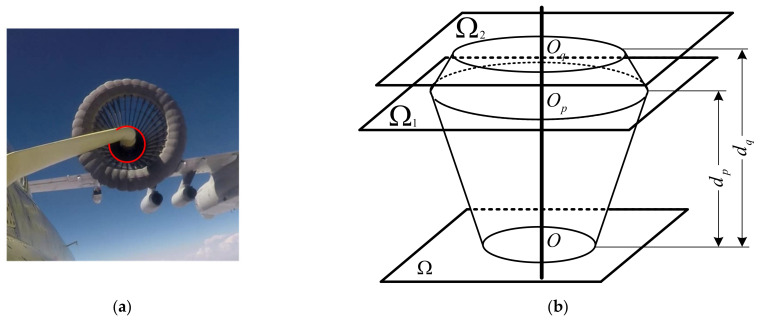
This is a figure of a drogue for aerial refueling. (**a**) The drogue during aerial refueling seen from the perspective of the receiver; (**b**) a side view of the drogue model.

In these scenes, the target with which visual navigation needs to deal is the drogue. The shape of the drogue shown in Figure 1 is approximately a BOR, and its outer contour is the surface of revolution (SOR), which contains many coaxial circular features. As the motion state of the tanker changes, the stabilizing umbrella of the drogue transitions between different opening and closing states. As a result, the actual size of most circular features is not fixed and its reference value cannot be obtained in advance. In this case, only the radius of the circle marked with the solid red line in Figure 1 is fixed, which is defined as the inner circle of the drogue in this paper and can be used directly to yield the analytical solution of position and attitude.

Nevertheless, the planes on which the circular features are located remain parallel, and the distance between the planes changes little. The vectors connecting the centers of circles are also collinear with the normal vector of each circle on the whole. Therefore, the coaxial constraints are proposed as follows:

1. There exists a structure of spatial circles on several planes parallel to the plane of the inner circle.
2. The centers of multiple spatial circles are collinear and the vectors composed of the circles’ center are collinear with the normal vector of the inner circle.

### 3.2. Proof

In this section, we prove that the constraints proposed in Section 3.1 can be adopted to eliminate the duality of the estimation of position and attitude. According to the two sets of solutions calculated by the inner circle and the distance between the planes of circles, two corresponding spatial structures can be restored in combination with the image. Denote $\Omega_{j}^{1}$ and $\Omega_{j}^{2}$ the *j*-th plane parallel to the inner circle corresponding to the two sets of solutions, and denote ${\overset{⌢}{O}}_{j}^{1}$ and ${\overset{⌢}{O}}_{j}^{2}$ the intersection curves between the space structures and the *j*-th plane parallel to the inner circle. If it can be proved that the space structure recovered from the false solution does not meet the coaxial constraints, that is, ${\overset{⌢}{O}}_{j}^{1}$ and ${\overset{⌢}{O}}_{j}^{2}$ are not circular at the same time, and the vectors composed of the circles’ center are not collinear with the normal vector of the inner circle, so the coaxial constraints can be used to eliminate the duality.

Without special instructions, the coordinates below are all in the camera coordinate system. Suppose that two sets of 5 DOF poses of the inner circle are calculated as $\left(O^{1},n^{1}\right)$ and $\left(O^{2},n^{2}\right)$ by Section 2.2. Among them, $O^{1}$ and $O^{2}$ are the candidate coordinates of the inner circle center, while $n^{1}$ and $n^{2}$ are the candidate normal vectors of the inner circle plane.

Two rotation matrices, denoted ${\bar{R}}_{1}$ and ${\bar{R}}_{2}$, were constructed by $n^{1}$ and $n^{2}$, respectively, which make the *z*-axis of the camera coordinate system parallel to the normal vector $n^{1}$ or $n^{2}$, and the *z*-axis coordinate of the target is positive. The coordinate system transforms as follows:

(7) $$\left\{\begin{array}{l} X_{1}={\bar{R}}_{1}X\\ X_{2}={\bar{R}}_{2}X \end{array}\right.$$

where X are the coordinates in the original camera coordinate system; $X_{1}$ and $X_{2}$ are in the transformed camera coordinate system. The relationships between them are as follows:

(8) $$X_{1}={\bar{R}}_{1}{\bar{R}}_{2}^{T}X_{2}=\bar{R}X_{2}$$

In accordance with the cause of duality, the camera’s optical center and the inner circle, denoted O, constitute an oblique cone space in the two transformed coordinate systems. The matrix expression of the oblique cone surface is given as follows:

(9) $$X_{i}{}^{T}\Phi_{i}X_{i}=X_{i}{}^{T}\left[\begin{array}{ccc} 1 & 0 & \alpha \\ 0 & 1 & \beta \\ \alpha & \beta & \gamma \end{array}\right]X_{i}=0,i=1,2$$

That is, the two transformed coordinate systems are constrained to the following formula:

(10) $$\left\{\begin{array}{l} X_{1}^{T}\Phi_{1}X_{1}=0\\ X_{2}^{T}\Phi_{2}X_{2}=0 \end{array}\right.$$

Substituting Equation (8) into Equation (10), we can obtain:

(11) $$\Phi_{2}={\bar{R}}^{T}\Phi_{1}\bar{R}$$

If the hypothesis that the dual solutions of the circle O make the two space structures of coaxial circle P satisfy the coaxial constraint (1) is not null, the camera’s optical center and circle P also form an oblique cone in the two transformed coordinate systems, $\Psi_{1}$ and $\Psi_{2}$, transformed by ${\bar{R}}_{1}$ and ${\bar{R}}_{2}$, that satisfies:

(12) $$\Psi_{2}={\bar{R}}^{T}\Psi_{1}\bar{R}$$

From the coaxial constraint (2), $\Psi_{1},\Phi_{1}$ and $\Psi_{2},\Phi_{2}$ satisfy the following formula:

(13) $$\left\{\begin{array}{l} \Phi_{1}=\Psi_{1}-\left[\begin{array}{ccc} 0 & 0 & \left(k-1\right)\alpha_{q1}\\ 0 & 0 & \left(k-1\right)\beta_{q1}\\ \left(k-1\right)\alpha_{q1} & \left(k-1\right)\beta_{q1} & \delta \gamma \end{array}\right]=\Psi_{1}-\Delta_{1}\\ \Phi_{2}=\Psi_{2}-\left[\begin{array}{ccc} 0 & 0 & \left(k-1\right)\alpha_{q2}\\ 0 & 0 & \left(k-1\right)\beta_{q2}\\ \left(k-1\right)\alpha_{q2} & \left(k-1\right)\beta_{q2} & \delta \gamma \end{array}\right]=\Psi_{2}-\Delta_{2} \end{array}\right.$$

where k≠1 is the ratio of the *z*-axis coordinates of circles O and P in the two transformed coordinate systems.

(14) $$\begin{array}{ccc} k=\frac{z_{q1}}{z_{p1}}=\frac{z_{q2}}{z_{p2}}=\frac{z_{q}}{z_{q}+z_{j}^{\left(w\right)}} & with & z_{q1}={\left(O^{1}\right)}^{T}\cdot n^{1},z_{q2}={\left(O^{2}\right)}^{T}\cdot n^{2} \end{array}$$

$z_{j}^{\left(w\right)}$ is the *z*-axis coordinate of P in the world coordinate system. From Equation (6), we can obtain: $z_{q1}=z_{q2}=z_{q}$. Denote

(15) $$\begin{array}{c} \bar{R}=\left[\begin{array}{ccc} r_{11} & r_{12} & r_{13}\\ r_{21} & r_{22} & r_{23}\\ r_{31} & r_{32} & r_{33} \end{array}\right]\\ \Delta_{1}=\left[\begin{array}{ccc} 0 & 0 & \alpha_{1}\\ 0 & 0 & \beta_{1}\\ \alpha_{1} & \beta_{1} & \gamma \end{array}\right]\\ \Delta_{2}=\left[\begin{array}{ccc} 0 & 0 & \alpha_{2}\\ 0 & 0 & \beta_{2}\\ \alpha_{2} & \beta_{2} & \gamma \end{array}\right] \end{array}$$

Combining Equation (11) with Equation (14), it can be inferred that:

(16) $$\Delta_{1}\bar{R}=\bar{R}\Delta_{2}$$

Substituting Equation (15) into Equation (16), Equation (16) can be written as:

(17) $$\begin{array}{cc} \; & \left[\begin{array}{ccc} \alpha_{1}r_{31} & \alpha_{1}r_{32} & \alpha_{1}r_{33}\\ \beta_{1}r_{31} & \beta_{1}r_{32} & \beta_{1}r_{33}\\ \alpha_{1}r_{11}+\beta_{1}r_{21}+\gamma r_{31} & \alpha_{1}r_{12}+\beta_{1}r_{22}+\gamma r_{32} & \alpha_{1}r_{13}+\beta_{1}r_{23}+\gamma r_{33} \end{array}\right]\\ \; & =\left[\begin{array}{ccc} \alpha_{2}r_{13} & \beta_{2}r_{13} & \alpha_{2}r_{11}+\beta_{2}r_{12}+\gamma r_{13}\\ \alpha_{2}r_{23} & \beta_{2}r_{23} & \alpha_{2}r_{21}+\beta_{2}r_{22}+\gamma r_{23}\\ \alpha_{2}r_{33} & \beta_{2}r_{33} & \alpha_{2}r_{31}+\beta_{2}r_{32}+\gamma r_{33} \end{array}\right] \end{array}$$

Analyzing the formula above, there exist the following situations:

1. $r_{13}=r_{31}=0,r_{23}=r_{32}=0$
  In this case, $r_{33}$ must be 1. There is a rotation transformation around the *z*-axis between the two coordinate systems with $\bar{R}$ as the rotation matrix, which means that the position and the normal vector of the circle are not changed. In other words, $\left(O^{1},n^{1}\right)$ and $\left(O^{2},n^{2}\right)$ are equal.
2. Other Situations

Comparing the left and right sides of Equation (17), we can obtain $\alpha_{1}=\alpha_{2}≙0$, $\beta_{1}=\beta_{2}≙0$, and γ≙0, where ≙ means possibly equal. Combining Equation (13) and Equation (17), we can obtain $\alpha_{q1}=\alpha_{q2},\beta_{q1}=\beta_{q2}$. If the intersecting curve is of the same radius, the following equation must exist.

(18) $$\left(\alpha_{q1}{}^{2}+\beta_{q1}{}^{2}-\gamma_{q1}\right)z_{q1}{}^{2}=r_{z}{}^{2}=\left(\alpha_{q2}{}^{2}+\beta_{q2}{}^{2}-\gamma_{q2}\right)z_{q2}{}^{2}$$

From Equation (18), we can obtain $\gamma_{q1}=\gamma_{q2}$. It can also be inferred that $\left(O^{1},n^{1}\right)$ are equal to $\left(O^{2},n^{2}\right)$.

In summary, the space structure recovered from the false analytical solution, which is caused by the duality of the pose estimation based on one circular feature, does not meet the coaxial constraints. Thereby, the coaxial constraints proposed by Section 3.1 can be utilized to eliminate the duality.

## 4. The Position and Attitude Estimation Method Based on the Coaxial Constraints

The Pseudo-algorithm of the position and attitude estimation method based on the coaxial constraints is shown in Algorithm 1.
**Algorithm 1:** Position and Attitude Estimation Algorithm Based on the Coaxial Constraints**Input:**  ***Image:*** *The parameters of ellipses have been fitted and matched*
  ***r:*** *Radius of inner circle*
  $z_{j}^{\left(w\right)}$***:*** *The *z*-axis coordinates of coaxial circles‘ center***Output:**  ***O:*** *The position of drogue in the camera coordinate system*  ***n*:** *The normal vector of drogue in the camera coordinate system*1: **function** COAXIALCIRCLEPOSE(***Image***, ***r***, $z_{j}^{\left(w\right)}$)2:   *Transform the parameters of ellipses into matrix expression*3:   *Calculate two sets of position and attitude of the inner circle*    $\left(O^{1},n^{1}\right)$,$\left(O^{2},n^{2}\right)$4:   **for**
*j = **1*** to ***J*** do5:    *Restore the spatial structures of*
$\left({\overset{⌢}{O}}_{j}^{1},{\overset{⌢}{O}}_{j}^{2}\right)$
*by*    $x_{*}^{\left(c\right)}=\frac{d}{\left(n^{T}\right)K^{-1}{\tilde{U}}_{*}}K^{-1}{\tilde{U}}_{*}$
6:   *Fit*
$\left\{O_{j}^{1},r_{j}^{1},\Delta r_{j}^{1}\right\}$
*and*
$\left\{O_{j}^{2},r_{j}^{2},\Delta r_{j}^{2}\right\}$
*from*
$\left({\overset{⌢}{O}}_{j}^{1},{\overset{⌢}{O}}_{j}^{2}\right)$7:   *Calculate*
$\left(O_{j}^{1},n_{j}^{1}\right)$
*and*
$\left(O_{j}^{2},n_{j}^{2}\right)$
*from*
$\left\{\begin{array}{l} r_{j}^{1},\begin{array}{ccc} & if & \Delta r_{j}^{1}<\Delta r_{j}^{2} \end{array}\\ r_{j}^{2},\begin{array}{ccc} & if & \Delta r_{j}^{1}>\Delta r_{j}^{2} \end{array} \end{array}\right.$8:   **end for** 9:   *Calculate*
$\phi^{1}$
*and*
$\phi^{2}$
*by*     $\phi^{i}=\sum_{j=1}^{J}\left(\frac{\Delta r_{j}^{i}}{r_{j}^{1}+r_{j}^{2}}\right)i=1,2$
10:   *Eliminate duality by*     $\left(O,n\right)=\left\{\begin{array}{l} \left(O^{1},n^{1}\right)\begin{array}{ccc} , & if & \phi^{1}<\phi^{2} \end{array}\\ \left(O^{2},n^{2}\right)\begin{array}{ccc} , & if & \phi^{1}>\phi^{2} \end{array} \end{array}\right.$
11:   *Eliminate duality by*     $\left(O,n\right)=\left\{\begin{array}{l} \left(O^{1},n^{1}\right)\begin{array}{ccc} , & if & {\left(\begin{array}{ccc} n_{j}^{1} & \mathrm{or} & n_{j}^{2} \end{array}\right)}^{T}n^{1}\approx 1 \end{array}\\ \left(O^{2},n^{2}\right)\begin{array}{ccc} , & if & {\left(\begin{array}{ccc} n_{j}^{1} & \mathrm{or} & n_{j}^{2} \end{array}\right)}^{T}n^{2}\approx 1 \end{array} \end{array}\right.$
12:   *Optimize normal vector by*     $\begin{array}{ccc} \hat{n}=\underset{\zeta }{\arg\min}\left(\sum_{j=1}^{J}{\left\|n_{j}^{\left(c\right)}-\zeta \right\|}^{2}+{\left\|n^{\left(c\right)}-\zeta \right\|}^{2}\right) & \mathrm{with} & \zeta^{T}\zeta =1 \end{array}$
13:   *Obtain translation vector by*     $\hat{t}=O$
14:   *result*
$\begin{array}{cc} \leftarrow & \hat{n},\hat{t} \end{array}$15:   **return** *result*16: **end function**

The algorithm uses the parameters of the ellipse, such as center coordinates, the major axis, the minor axis, the inclination angle, the radius of the inner circle, and the *z*-axis coordinates of the inner coaxial circles in the world coordinate system as input.

First of all, the matrix expression of ellipses from the input parameters of the ellipses and the position and attitude with the known condition of the inner circle are calculated by the algorithm in Section 2.2. Then, two corresponding spatial structures are restored using the method described in Section 4.1. The method described in Section 4.2 is used to eliminate the duality. Finally, the normal vector of the target is optimized by fusing multiple circular features in the spatial structure.

### 4.1. Spatial Structure Recovery

**Figure 2:**
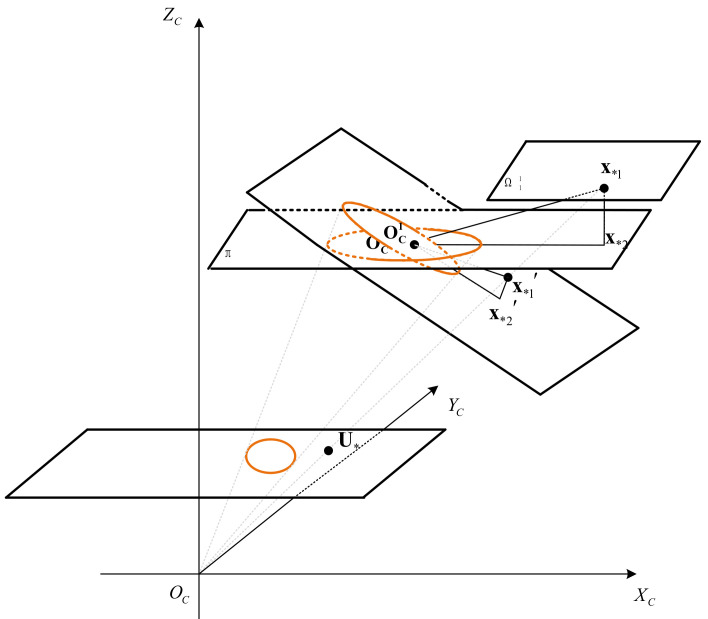
This is a figure of the spatial relationship of a point and circle corresponding to the duality.

The spatial relationship of a 3D point and circle corresponding to the duality is shown in Figure 2. Take the coaxial circle corresponding to $\left(O^{1},n^{1}\right)$ as an example, which is on the plane parallel to the inner circle, and the *z*-axis coordinate is $z_{j}^{\left(w\right)}$ in the world coordinate system. The restored 3D point $x_{*}^{\left(c\right)}={\left(x^{*},y^{*},z^{*}\right)}^{T}$ on the coaxial circle is the intersection of $\vec{O_{c}U_{*}}$ (the vector connecting the camera’s optical center $O_{c}$ and the imaging point $U_{*}$) with the space plane, where the coaxial circle is located. $x_{*}^{\left(c\right)}$ satisfies the following:

(19) $$\left\{\begin{array}{l} z^{*}{\tilde{U}}_{*}=Kx_{*}^{\left(c\right)}\\ {\left(n^{1}\right)}^{T}x_{*}^{\left(c\right)}-d=0 \end{array}\right.$$

where $d=\frac{\left\|{\left(n^{1}\right)}^{T}O^{1}\right\|}{\left\|n^{1}\right\|}+z_{j}^{\left(w\right)}$, according to the relationship between the two planes. It can be inferred that:

(20) $$x_{*}^{\left(c\right)}=\frac{d}{{\left(n^{1}\right)}^{T}K^{-1}{\tilde{U}}_{*}}K^{-1}{\tilde{U}}_{*}$$

Select N points on the curve uniformly and restore the coordinates of the 3D points on the plane $\Omega_{1}$ in terms of Equation (20). Denote the N 3D points, the center of the coaxial circle, and the midpoint of any two different points as $x_{*}^{\left(c\right)}{}_{i}={\left(x_{i}^{*},y_{i}^{*},z_{i}^{*}\right)}^{T}$, $O_{*}={\left(x_{o}^{*},y_{o}^{*},z_{o}^{*}\right)}^{T}$, and $x_{*}^{\left(c\right)}{}_{ij}={\left(\frac{x_{i}^{*}+x_{j}^{*}}{2},\frac{y_{i}^{*}+y_{j}^{*}}{2},\frac{z_{i}^{*}+z_{j}^{*}}{2}\right)}^{T}$, respectively, where i,j=1,2,…N. Since the center of a circle must be on the intersection line of the mid vertical plane of any two points, the vector $\vec{O_{1}x_{*}^{\left(c\right)}{}_{ij}}$ should be perpendicular to $\vec{x_{*}^{\left(c\right)}{}_{i}x_{*}^{\left(c\right)}{}_{j}}$. This means that:

(21) $$\left(x_{j}^{*}-x_{i}^{*},y_{j}^{*}-y_{i}^{*},z_{j}^{*}-z_{i}^{*}\right){\left(\frac{x_{i}^{*}+x_{j}^{*}}{2}-x_{o}^{*},\frac{y_{i}^{*}+y_{j}^{*}}{2}-y_{o}^{*},\frac{z_{i}^{*}+z_{j}^{*}}{2}-z_{o}^{*}\right)}^{T}=0$$

After sorting out the equation, we can obtain

(22) $$\Delta x_{ij}^{*}\cdot x_{o}^{*}+\Delta y_{ij}^{*}\cdot y_{o}^{*}+\Delta z_{ij}^{*}\cdot z_{o}^{*}=c_{ij}$$

where $\Delta x_{ij}^{*}=x_{i}^{*}-x_{j}^{*}$, $\Delta y_{ij}^{*}=y_{i}^{*}-y_{j}^{*}$, $\Delta z_{ij}^{*}=z_{i}^{*}-z_{j}^{*}$, and $c_{ij}=\frac{{\left(x_{i}^{*}\right)}^{2}+{\left(y_{i}^{*}\right)}^{2}+{\left(z_{i}^{*}\right)}^{2}-{\left(x_{j}^{*}\right)}^{2}-{\left(y_{j}^{*}\right)}^{2}-{\left(z_{j}^{*}\right)}^{2}}{2}$. N-1 linearly independent vectors, denoted ${\left(\Delta x_{ij}^{*},\Delta y_{ij}^{*},\Delta z_{ij}^{*}\right)}^{T}$, can be obtained from the N 3D points, and the following formula can be obtained:

(23) $$\left[\begin{array}{ccc} \Delta x_{12}^{*} & \Delta y_{12}^{*} & \Delta z_{12}^{*}\\ \Delta x_{23}^{*} & \Delta y_{23}^{*} & \Delta z_{23}^{*}\\ \ldots & \ldots & \ldots \\ \Delta x_{N-1N}^{*} & \Delta y_{N-1N}^{*} & \Delta z_{N-1N}^{*} \end{array}\right]{\left[\begin{array}{ccc} x_{o}^{*} & y_{o}^{*} & z_{o}^{*} \end{array}\right]}^{T}=\left[\begin{array}{c} c_{1}\\ c_{2}\\ \ldots \\ c_{N-1} \end{array}\right]$$

Rewrite Equation (23) as:

(24) $$AO_{*}=C$$

Since the circle’s center must be on $\Omega_{1}$, which can be represented by $n_{1}$ (the normal vector) and d (the intercept), we can define the error equation as:

(25) $$\begin{array}{ccc} f\left(O_{*}\right)={\left\|AO_{*}-C\right\|}^{2}+\lambda \left(BO_{*}-1\right) & with & B=n_{1}/d \end{array}$$

The coordinates of the circle’s center are the least square solution of the above equation.

(26) $$\left[\begin{array}{c} O_{*}\\ \lambda \end{array}\right]=\left[\begin{array}{cc} A^{T}A & B^{T}\\ B & 0 \end{array}\right]\left[\begin{array}{c} A^{T}C\\ 1 \end{array}\right]$$

Furthermore, the radius and the roundness of the circle are obtained by Equation (27). We define the roundness of the circle, denoted $\Delta r^{*}$, as the average roundness of the points.

(27) $$\left\{\begin{array}{l} l_{i}=\sqrt{{\left(x_{i}^{*}-x_{o}^{*}\right)}^{2}+{\left(y_{i}^{*}-y_{o}^{*}\right)}^{2}+{\left(z_{i}^{*}-z_{o}^{*}\right)}^{2}}\\ r=\frac{1}{N}\sum_{i=1}^{N}l_{i}\\ \Delta r=\sum_{i=1}^{N}\left|l_{i}-r\right| \end{array}\right.$$

The radius and the roundness of other coaxial circles can also be calculated.

### 4.2. Elimination of Duality

As the name suggests, the two-level coaxial constraints algorithm consists of two parts. The first level is the coaxial constraint (1) in Section 3.1, that is, the elimination of the duality by using the roundness of the spatial structure. After restoring the spatial structures by Section 4.1, we can obtain the radius and roundness of the coaxial circles, denoted $r_{j}^{1},r_{j}^{2}$ and $\Delta r_{j}^{1},\Delta r_{j}^{2}$, respectively, corresponding to the two pose solutions of the inner circle. If there are J coaxial circles, the definition is as follows:

(28) $$\begin{array}{ccccc} \phi^{i}=\sum_{j=1}^{J}\left(\frac{\Delta r_{j}^{i}}{r_{j}^{1}+r_{j}^{2}}\right) & with & i=1,2 & and & j=1,2\ldots J \end{array}$$

Then, the first-level constraint can be expressed as the following equation.

(29) $$\left(O,n\right)=\left\{\begin{array}{l} \left(O^{1},n^{1}\right)\begin{array}{ccc} & if & \phi^{1}<\phi^{2} \end{array}\\ \left(O^{2},n^{2}\right)\begin{array}{ccc} & if & \phi^{1}>\phi^{2} \end{array} \end{array}\right.$$

The second-level constraint is the coaxial constraint (2) in Section 3.1, that is, the normal vectors of multiple planes where the circles are located are parallel. Using the radius calculated by Equation (27), two sets of positions and attitudes of multiple coaxial circles, denoted $\left(O_{j}^{1},n_{j}^{1}\right)$ and $\left(O_{j}^{2},n_{j}^{2}\right)$, respectively, can be solved for. The second-level coaxial constraint can be written as:

(30) $$\left(O,n\right)=\left\{\begin{array}{l} \left(O^{1},n^{1}\right)\begin{array}{ccc} & if & {\left(\begin{array}{ccc} n_{j}^{1} & \mathrm{or} & n_{j}^{2} \end{array}\right)}^{T}n^{1}\approx 1 \end{array}\\ \left(O^{2},n^{2}\right)\begin{array}{ccc} & if & {\left(\begin{array}{ccc} n_{j}^{1} & \mathrm{or} & n_{j}^{2} \end{array}\right)}^{T}n^{2}\approx 1 \end{array} \end{array}\right.$$

After the coaxial constraints are applied, the normal vectors of the planes where multiple circles are located can also be obtained.

### 4.3. Fusion of Multiple Circular Features

After the two-level coaxial constraints described in the previous subsection are applied, the center position of the inner circle and the normal vectors of multiple planes can be obtained. In the pose fusion problem of multiple coaxial circles with a known radius, there are the following relationships among the circles.

(31) $$O_{j}^{\left(c\right)}=z_{j}{}^{\left(w\right)}n^{\left(c\right)}+t^{\left(c\right)}$$

where $z_{j}{}^{\left(w\right)}$ is the *z*-axis coordinate of $O_{j}$ in the world coordinate system. Let $\begin{array}{ccc} O_{jk}^{\left(c\right)}=O_{j}^{\left(c\right)}-O_{k}^{\left(c\right)} & with & j,k\in \left\{1,2\ldots J\right\} \end{array}$, where $O_{j}^{\left(c\right)}$ and $O_{k}^{\left(c\right)}$ are the centers of the coaxial circles obtained by Section 4.2, then

(32) $$O_{jk}^{\left(c\right)}=(z_{j}^{\left(w\right)}-z_{k}^{\left(w\right)})n^{\left(c\right)}$$

In Reference [29], it was shown that the pose fusion algorithm for multiple coaxial circles with a known radius is equivalent to solving the following equation.

(33) $$\begin{array}{ccc} \hat{n}=\underset{\zeta }{\arg\min}\left(\sum_{j=1}^{J}{\left\|n_{j}^{\left(c\right)}-\zeta \right\|}^{2}+\sum_{j,k\in \left\{1,2\ldots J\right\}}{\left\|\frac{O_{jk}^{\left(c\right)}}{z_{j}^{\left(w\right)}-z_{k}^{\left(w\right)}}-\zeta \right\|}^{2}\right) & with & \zeta^{T}\zeta =1 \end{array}$$

The analysis of Equation (6) shows that the radius estimation error has little influence on the error of $n_{j}^{\left(c\right)}$ but some influence on the error of $O_{j}^{\left(c\right)}$. When the value of $\left|z_{j}^{\left(w\right)}-z_{k}^{\left(w\right)}\right|$ is small, the vector $\frac{O_{jk}^{\left(c\right)}}{z_{j}^{\left(w\right)}-z_{k}^{\left(w\right)}}$ has a little error. Accordingly, we use the following formula to solve $\hat{n}$.

(34) $$\begin{array}{ccc} \hat{n}=\underset{\zeta }{\arg\min}\left(\sum_{j=1}^{J}{\left\|n_{j}^{\left(c\right)}-\zeta \right\|}^{2}+{\left\|n^{\left(c\right)}-\zeta \right\|}^{2}\right) & \mathrm{with} & \zeta^{T}\zeta =1 \end{array}$$

where $n_{j}^{\left(c\right)}$ and $n^{\left(c\right)}$ are the normal vectors of the coaxial circle and the inner circle solved by Section 4.2. Considering that the error of $O_{j}$ is a little larger, the solution for the position of the inner circle is selected as the corresponding $\hat{t}$.

So far, the position and the normal vector of the target with coaxial constraints have been solved.

## 5. Results and Analysis

### 5.1. Evaluation Indices

The simulations of and experiments on the drogue model were designed to verify the effectiveness of the proposed algorithm. The performance of the algorithm was evaluated in terms of two aspects: the duality elimination success rate and the accuracy of the normal vector of the target.

Assuming that the position and the normal vector of the target calculated by our algorithm were $\left(\hat{n},\hat{t}\right)$ and the benchmarks we set were $\left(R_{g},t_{g}\right)$, the error was defined to be composed of the angle error Δθ (the angle between the solved normal vector and the ground truth $r_{3}$, where $r_{3}$ is the third column of the matrix $R_{g}$) and the translation error Δt.

(35) $$\begin{array}{l} \Delta \theta =\arccos\left({\hat{n}}^{T}r_{3}\right)\\ \Delta t=\left\|\hat{t}-t_{g}\right\| \end{array}$$

The evaluation criterion of the successful elimination of the duality was that the angle error and the translation error of $\left(\hat{n},\hat{t}\right)$ were less than what was eliminated.

In this paper, the success rate of duality elimination was defined as the percentage of frames with successful elimination of the duality with respect to the total number of image frames.

### 5.2. Simulations

The simulations were carried out on a simulation system based on MATLAB, in which the resolution was 1600 × 1200 pixels, the pixel size was 0.01 × 0.01 mm, the focal distance was 8 mm, and the principal point of the photograph was (800.00, 600.00).

To verify the anti-interference ability and the robustness to relative distances of the algorithm, we carried out comparative simulations under different conditions, including noise in the image feature (an error in fitting the feature’s parameters), noise in the spatial structure (deformation of the drogue’s frame), and different relative distances between the camera and the target.

The world coordinate system was established with the inner circle’s center as the origin and the *z*-axis perpendicular to the plane of the inner circle and facing away from the camera.

The parameters of the target were set according to the measurements of the actual drogue. The radius of the inner circle was r=125.0 mm, and the coordinates of the inner circle’s center were $\left(0,0,0\right)$; the radius of the circle $O_{1}$ was $r_{1}=385.0$ mm, and the coordinates of the circle’s center were $\left(0,0,-210\right)$; the radius of the circle $O_{2}$ was $r_{2}=320.0$ mm, and the coordinates of the circle’s center were $\left(0,0,-310\right)$. Only $O_{2}$ was used in the duality elimination simulations.

1. Elimination of Duality

- Noise in the Image Feature

**Figure 3:**
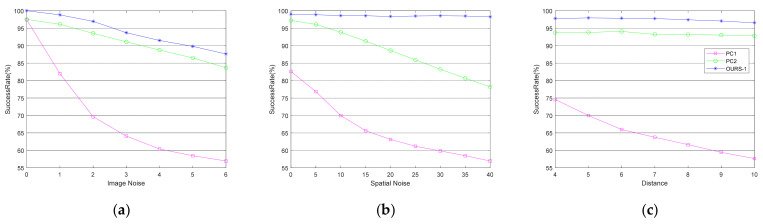
This is a figure showing the success rate of the algorithm under different simulation conditions. The curves corresponding to the algorithm in References [18,20] are marked as PC1 and PC2. The coordinates of the two points were set to (300, 300, 0) and (150, 150, −500), and gaussian noise with a mean of 0 and a variance of 5 was added to the position. (**a**) The success rate of the algorithms under different levels of noise in the image; (**b**) The success rate of the algorithms under different levels of noise in the spatial structure; (**c**) The success rate of the algorithms under different *z*-axis coordinates of the targets.

The *z*-axis coordinate of the target in the camera coordinate system was 5 m, and the *x*-axis coordinate, *y*-axis coordinate, heading, pitch, and roll were set as a random value within the range of ±1.5 m, ±1.5 m, ±30°, ±30°, and ±15°, respectively. We added white gaussian noise with a mean value of 0 and a variance of 10 to the center coordinates of $O_{2}$, and noise with a mean value of 0 and a variance of 20 to $r_{2}$. The noise in the image features was set as white gaussian noise with a mean value of 0 and a variance of interval 1 from 1 to 6. According to the above simulation conditions, images of the target were generated, and the features in the image with the noise disturbance were extracted to solve for the position and the normal vector. Each test generated 2000 simulated images to run the algorithm, and the results of each simulation condition are the average of 10 tests. The results are shown in Figure 3.

- Noise in the Spatial Structure

The setting of the target’s pose in the camera coordinate system was the same as above. We added white gaussian noise with a mean of 0 and a variance of 2 to the image features. At this time, the noise in the coordinates of $O_{2}$ was set as white gaussian noise with a mean value of 0 and a variance of interval 5 from 0 to 40. The noise in $r_{2}$ was twice the noise in $O_{2}$. Under the above conditions, each test generated 2000 simulated images to run the algorithm, and the results of each simulation condition are an average of 10 tests. The results are shown in Figure 3b.

- Different *z*-axis Coordinates of Targets

We added white gaussian noise with a mean of 0 and a variance of 2 to the image features, white gaussian noise with a mean value of 0 and a variance of 20 to $O_{2}$, and white gaussian noise with a mean value of 0 and a variance of 40 to $r_{2}$. The settings of the *x*-axis coordinate, *y*-axis coordinate, and attitude were the same as above, but the *z*-axis coordinates were 4–10 m with an interval of 1. Under the above conditions, each test generated 2000 simulated images to run the algorithm, and the results of each simulation condition are an average of 10 tests. The results are shown in Figure 3c.

To verify the effectiveness of our algorithm and consider its applicability to drogues for air refueling, we selected the algorithms in References [18,20] for comparison, which are the curves marked as PC1 and PC2, respectively, in Figure 3.

The results in Figure 3a show that the proposed algorithm for duality elimination has a good anti-interference ability against the noise in the image features. It also has excellent performance when there is a large disturbance. That is, when the variance is 5, the success rate can be higher than 90%. The algorithm in Reference [20] also has a high degree of robustness to the noise in the image features, but it is about 3% lower than ours.

The results in Figure 3b demonstrate that the proposed algorithm is surprisingly robust to the noise in the spatial structure. Under noise with a variance of 40, the algorithm in Reference [18] almost fails, but our success rate is higher than 98.5%, which also has obvious advantages compared with the 78.20% of the algorithm in Reference [20].

This shows that the performance of our algorithm remains pretty consistent with changes in the radius of the coaxial circle and the distance between the planes caused by the opening and closing of the drogue’s stabilizing umbrella during aerial refueling.

It is worth mentioning that there is about a 30% difference between the results of the different simulations using the algorithms in References [18,20]. This means that the two algorithms are sensitive to the pose of the target.

The results in Figure 3c show that our algorithm is effective when different *z*-axis coordinates of the target within 10 m are used. Moreover, it can obtain a higher success rate (about 6% higher) than the algorithm in [20] under the same conditions.

2. Accuracy of the Normal Vector

**Figure 4:**
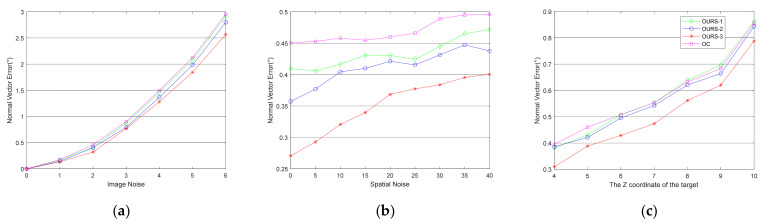
This is a figure about the angle error of the normal vector under different simulation conditions. The normal vector that only eliminates duality by $O_{1}$ and $O_{2}$ is marked as OC, and the normal vectors solved by the fusion algorithm with $O_{1}$, $O_{2}$ and both $O_{1}$ and $O_{2}$ are marked as OURS-1, OURS-2, and OURS-3, respectively. (**a**) The angle error of the normal vector under different levels of noise in image features; (**b**) The angle error of the normal vector under different levels of noise in the spatial structure; (**c**) The angle error of the normal vector under different *z*-axis coordinates of the targets.

**Figure 5:**
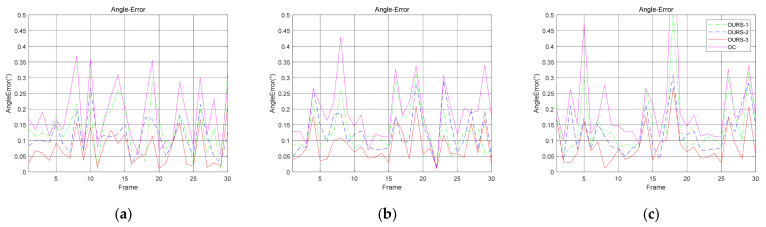
This is a figure showing some examples of simulations for the angle error of the normal vector. (**a**) The angle error of the normal vector when the variance in the image feature noise is 2; (**b**) The angle error of the normal vector when the variance in the spatial structure noise is 20; (**c**) The angle error of the normal vector at *z*-axis coordinates of 6 m.

We also evaluated the accuracy of the normal vector solved by the algorithm for the fusion of multiple circular features under the same simulation conditions as above. We compared the accuracy of the normal vectors solved by the different algorithms in simulations. The results are shown in Figure 4 and Figure 5.

Comparing the curves marked OURS-1, OURS-2, OURS-3, and OC in Figure 4a–c, it is noticeable that fusing multiple coaxial circles can effectively improve the accuracy of the normal vector. The mean value of the curves for each simulation is shown in Table 1. From the curves OC and OURS-3, the data show that our fusion algorithm with both O1 and O2 can effectively improve the accuracy of the target’s normal vector. The advantages are quite obvious since the angle errors of the normal vector solved by the fusion algorithm with both O1 and O2 achieve good results. In addition, the angle errors corresponding to the simulations of different levels of image feature noise and spatial structure noise and different *z*-axis coordinates are reduced by 18.4%, 25.6%, and 15.4%, respectively, compared with the result that only eliminates duality by O1 and O2. Once again, these results show that our algorithm is robust against the noise in the image features and spatial structure and is effective when different *z*-axis coordinates of the target within at least 10 m are used. The normal vector solved by the fusion algorithm with O1 and O2 (marked as OURS-1 and OURS-2, respectively) can also improve the accuracy. However, using two coaxial circles is much better than using one. Moreover, the results for O1 and O2 are different in the case of a single coaxial circle. This is discussed in the next section.

Notice that the curve of OC is under the curves of OURS-1 in Figure 4c when the *z*-axis coordinates of the target are greater than 8 m. The results here are due to the success rate of duality elimination with two coaxial circles being higher than that with one coaxial circle and the pose estimation algorithm in Section 2.1 providing a highly accurate solution with less image feature noise.

3. Influence of spatial structure on the algorithm’s performance

The striking result that emerges from the curves of OURS-1 and OURS-2 in Figure 4 is that there are great differences between them. To verify the influence of different spatial structures of the coaxial circles on the algorithm, evaluation simulations were carried out.

White gaussian noise with a mean of 0 and a variance of 2 was added to the image features. Noise with a mean value of 0 and a variance of 20 was added to the position of $O_{1}$, and noise with a mean value of 0 and a variance of 30 was added to the position of $r_{1}$. The settings of the *x*-axis coordinate, *y*-axis coordinate, and attitude were the same as above.

**Figure 6:**
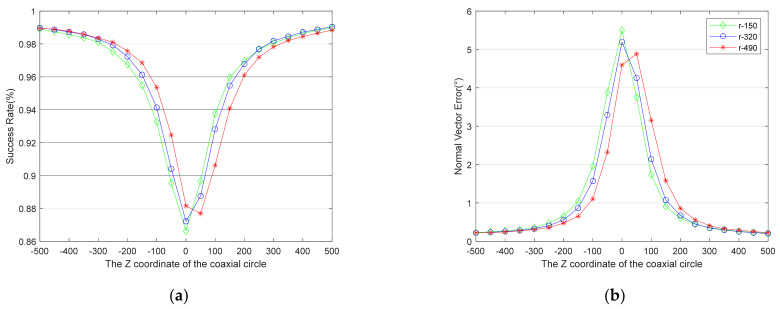
This is a figure about the relationship between the performance of the proposed algorithm and the target’s spatial structure when the *z*-axis coordinate of the target is 5 m. The curves of the different radii are marked r-150, r-320, and r-490, respectively. (**a**) The curves of the success rate. (**b**) The curves of the normal vector’s angle error.

**Figure 7:**
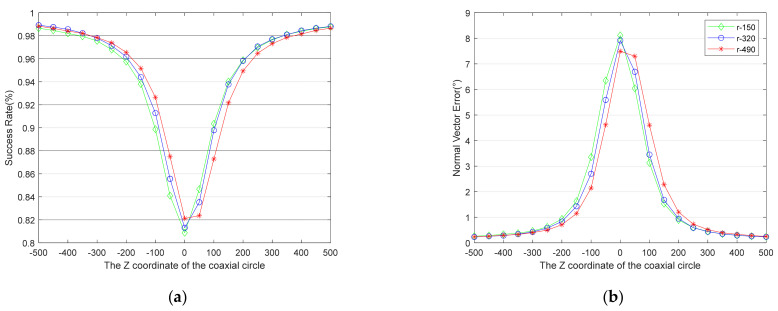
This is a figure about the relationship between the performance of the proposed algorithm and the target’s spatial structure when the *z*-axis coordinate of the target is 7 m. (**a**) The curves of the success rate. (**b**) The curves of the normal vector’s angle error.

**Figure 8:**
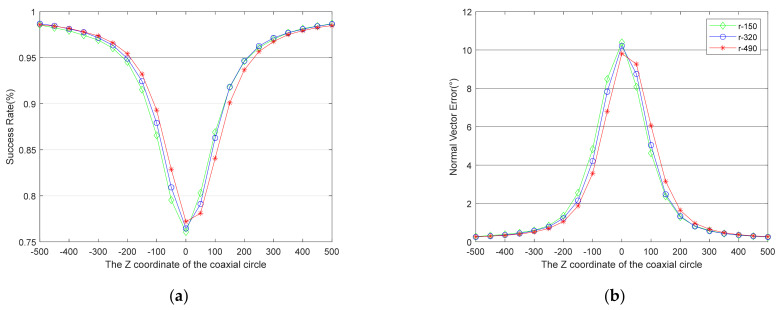
This is a figure about the relationship between the performance of the proposed algorithm and the target’s spatial structure when the *z*-axis coordinate of the drogue is 9 m. (**a**) The curves of the success rate. (**b**) The curves of the normal vector’s angle error.

Under the above simulation conditions, comparative simulations of different *z*-axis coordinates of the target (5 m, 7 m, 9 m), different radii of the coaxial circles (150 mm, 320 mm, 490 mm), and different center coordinates (50 mm intervals within ±500) were carried out. Each test generated 2000 simulated images to run the algorithm, and the results of each simulation condition are an average of 10 tests. The results are shown in Figure 6, Figure 7, Figure 8.

The relationship between the success rate and the target’s spatial structure is presented in Figure 6a, Figure 7a, Figure 8a, while the relationship between the accuracy of the normal vector and the target’s spatial structure is presented in Figure 6b, Figure 7b, Figure 8b. All the curves show that the absolute values of the coaxial circles’ *z*-axis coordinates in the world coordinate system are smaller, and the performance of the algorithm is worse. Moreover, all the curves are not completely symmetrical when the *z*-axis coordinates of the target in the camera coordinate system are negative and positive. When the absolute values of the *z*-axis coordinates of the coaxial circle with the same radius in the world coordinate system are equal, the negative one is better than the positive one, e.g., the points with abscissa of −100 and 100 on the curve marked r-490 in Figure 6, Figure 7, Figure 8.

More interestingly, Figure 6, Figure 7, Figure 8 reveal that the relationship between the performance of the proposed algorithm and the radii of the coaxial circles is the opposite when the *z*-axis coordinates of the coaxial circle in the world coordinate system are positive and negative. When the *z*-axis coordinates of the coaxial circle are negative, the coaxial circle with a radius of 490 mm (r-490) has a better success rate and the accuracy of the normal vector is higher. In contrast, the coaxial circle with a radius of 150 mm (r-150) has better performance when the *z*-axis coordinates of the coaxial circle are positive.

All in all, the results reveal a relationship between the performance of the proposed algorithm and the spatial structure of the target, and the algorithm’s performance is not consistent when the *z*-axis coordinates of the drogue in the camera coordinate system are different. Coaxial circles with a reasonable structure could greatly improve the performance of the algorithm. The following conclusions can be drawn by comparing the curves in the figure:

- The farther away the plane of the coaxial circle is from the plane of the inner circle, the better the performance of the algorithm is. It would be a good choice to select a circular feature in the plane that is more than twice the radius of the inner circle away from the plane of the inner circle. (When the two planes are very close, the false solutions of the two circles are also very similar, which leads to a reduction in the success rate of duality elimination.)
- When the distance between the plane of the coaxial circle and the plane of the inner circle is the same, the closer the coaxial circle plane is to the camera, the better the performance of the algorithm is.
- When the plane of the coaxial circle is closer to the camera than the inner circle, the larger the radius of the coaxial circle is, the better the performance of the algorithm is.
- When the plane of the coaxial circle is farther away from the camera than the inner circle, the smaller the radius of the coaxial circle is, the better the performance of the algorithm is.

### 5.3. Experiments on the Drogue Model

**Figure 9:**
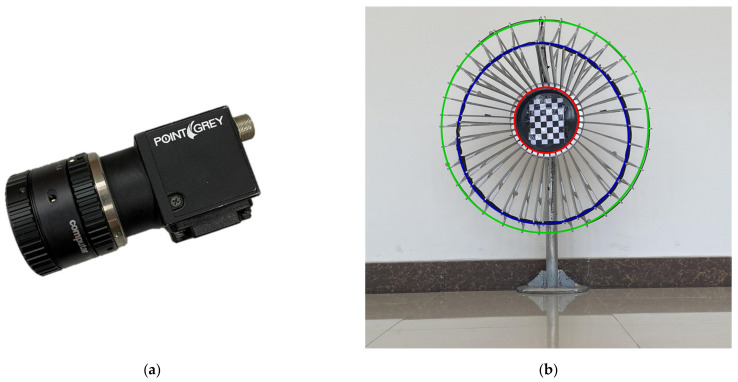
This is a figure about the experimental equipment. (**a**) A photo of the camera and the lens used for image capture. (**b**) A photo of the drogue model.

The proposed algorithm was tested on a sequence of 49 images captured by the Point Grey BFLY-U3-23S6C-C camera shown in Figure 9a with the drogue model shown in Figure 9b. The image resolution was set to 960 × 600 pixels. The focal length of the camera was calibrated as follows: fu = 1343.44, fv = 1347.53, principle point u_0_ = 493.53, v_0_ = 289.09.

The drogue model was made according to a real drogue, in which the diameter of the inner circle marked with a red solid line is 250 mm. The origin of the world coordinate system of the target was located at the center of the inner circle, and the *z*-axis was perpendicular to the plane and faced away from the camera.

The coordinates of the other coaxial circles were $O_{p}=(0,0,-210)$ and $O_{q}=(0,0,-310)$. To simulate the deformation of the drogue during aerial refueling, an image of the drogue model was captured with some stretching and compression.

The ground truth corresponding to each frame was obtained by fusing and optimizing the pose of the target with a chessboard in multiple images according to the method described in [13].

Experiments were carried out with circle $O_{p}$, circle $O_{q}$, and both, which are marked O1, O2, and O3, respectively. The success rate of duality elimination is presented in Table 2, which shows that our algorithm for duality elimination has high practicability since it succeeded in all the images captured in the experiment.

**Figure 10:**
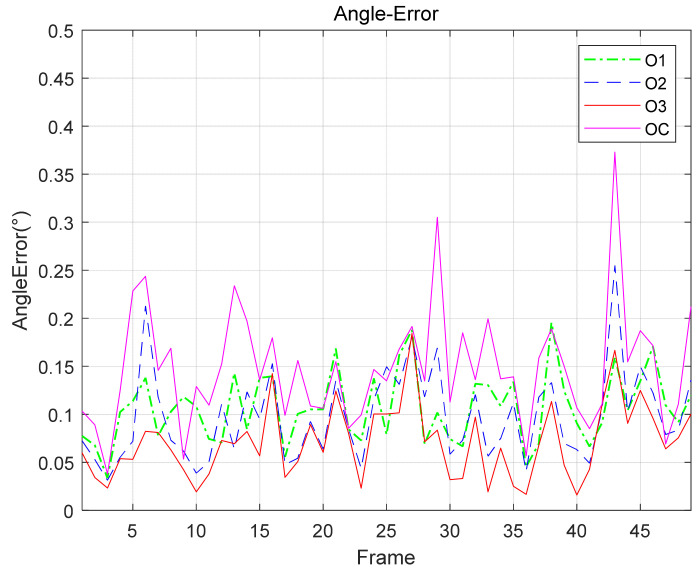
This is a figure of the angle error of the normal vector corresponding to the captured images.

The angle error of the normal vector is shown in Figure 10, where the curve marked OC describes the normal vector’s angle error with the duality eliminated by both $O_{p}$ and $O_{q}$ without fusion. The mean value of the curves O1, O2, O3, and OC is 0.11°, 0.10°, 0.08°, and 0.15°, respectively.

Furthermore, to test the computational efficiency of the algorithm, points of interval 10 from 20 to 60 were selected to recover the spatial structures by Section 4.1. The case of one coaxial circle was compared with the case of two coaxial circles. 

The relationship between the computation time and the number of points for the recovery of spatial structures is presented in Table 3. As the number of points increases, the computation time of the algorithm increases. In the experiments corresponding to Table 2 and Figure 10, 30 points were selected for the spatial structure recovery of each circle, and the corresponding computation time is shown (red font) in Table 3. The case of one coaxial circle is 1.2 ms while that of two coaxial circles is 2.1 ms. This result shows that our algorithm owns a desirable real-time performance.

Overall, these results suggest that our proposed algorithm is capable of solving the problem of estimating the pose of the drogue, which means that it can effectively eliminate duality and simultaneously improve the accuracy of the target’s normal vector in real time.

## 6. Conclusions

In summary, we have proposed a monocular visual position and attitude estimation method of a drogue based on coaxial constraints, which can effectively eliminate the duality in pose estimation by a single circular feature and greatly improve the accuracy of the optimized normal vector. The effectiveness and robustness of the method were verified by simulations and experiments. Furthermore, we established a basis for engineering through a number of simulations and experiments. To our knowledge, no one has so far explored the relationship between the algorithm’s performance and the target’s spatial structures.

## Figures and Tables

**Table 1:** This is a table about the mean error of the normal vector in the curves under different simulation conditions.

Curves	Corresponding to Simulation (a)	Corresponding to Simulation (b)	Corresponding to Simulation (c)
Ours-1	0.13	0.13	0.15
Ours-2	0.11	0.12	0.12
Ours-3	0.07	0.08	0.08
OC	0.17	0.19	0.20

**Table 2:** The success rate of the algorithm with different coaxial circles.

Circles	Success Rate (%)
O1	100
O2	100
O3	100

**Table 3:** This is a table of the relationship between the computation time and the number of points for spatial structure recovery.

Number of Points for Each Circle	Computation Time of 1 Coaxial Circle (ms)	Computation Time of 2 Coaxial Circles (ms)
20	0.9	1.3
30	1.2	2.1
40	1.4	2.6
50	1.6	3.1
60	1.9	3.8
